# Spatially resolved metabolic distribution for unraveling the physiological change and responses in tomato fruit using matrix-assisted laser desorption/ionization–mass spectrometry imaging (MALDI–MSI)

**DOI:** 10.1007/s00216-016-0118-4

**Published:** 2016-12-08

**Authors:** Junya Nakamura, Tomomi Morikawa-Ichinose, Yoshinori Fujimura, Eisuke Hayakawa, Katsutoshi Takahashi, Takanori Ishii, Daisuke Miura, Hiroyuki Wariishi

**Affiliations:** 1 Graduate School of Bioresource and Bioenvironmental Sciences, Kyushu University, 6-10-1 Hakozaki, Higashi-ku, Fukuoka, 812-8581 Japan; 2 Innovation Center for Medical Redox Navigation, Kyushu University, 3-1-1 Maidashi, Higashi-ku, Fukuoka, 812-8582 Japan; 3 Okinawa Institute of Science and Technology Graduate University, 1919-1 Tancha, Onna-son, Kunigami-gun, Okinawa, 904-0495 Japan; 4 National Institute of Advanced Industrial Science and Technology, 2-41-6 Aomi, Koto-ku, Tokyo, 135-0064 Japan; 5 Bio-architecture Center, Kyushu University, 6-10-1 Hakozaki, Higashi-ku, Fukuoka, 812-8581 Japan; 6 Faculty of Arts and Sciences, Kyushu University, 744 Motooka, Nishi-ku, Fukuoka, 819-0395 Japan

**Keywords:** Localization, MALDI–MSI, Metabolic alterations, Physiological changes and responses, Tomato fruit (*Solanum lycopersicum* L.)

## Abstract

Information on spatiotemporal metabolic behavior is indispensable for a precise understanding of physiological changes and responses, including those of ripening processes and wounding stress, in fruit, but such information is still limited. Here, we visualized the spatial distribution of metabolites within tissue sections of tomato (*Solanum lycopersicum* L.) fruit using a matrix-assisted laser desorption/ionization–mass spectrometry imaging (MALDI–MSI) technique combined with a matrix sublimation/recrystallization method. This technique elucidated the unique distribution patterns of more than 30 metabolite-derived ions, including primary and secondary metabolites, simultaneously. To investigate spatiotemporal metabolic alterations during physiological changes at the whole-tissue level, MALDI–MSI was performed using the different ripening phenotypes of mature green and mature red tomato fruits. Although apparent alterations in the localization and intensity of many detected metabolites were not observed between the two tomatoes, the amounts of glutamate and adenosine monophosphate, umami compounds, increased in both mesocarp and locule regions during the ripening process. In contrast, malate, a sour compound, decreased in both regions. MALDI–MSI was also applied to evaluate more local metabolic responses to wounding stress. Accumulations of a glycoalkaloid, tomatine, and a low level of its glycosylated metabolite, esculeoside A, were found in the wound region where cell death had been induced. Their inverse levels were observed in non-wounded regions. Furthermore, the amounts of both compounds differed in the developmental stages. Thus, our MALDI–MSI technique increased the understanding of the physiological changes and responses of tomato fruit through the determination of spatiotemporally resolved metabolic alterations.

**Electronic supplementary material:**

The online version of this article (doi:10.1007/s00216-016-0118-4) contains supplementary material, which is available to authorized users.

**Graphical abstract:**
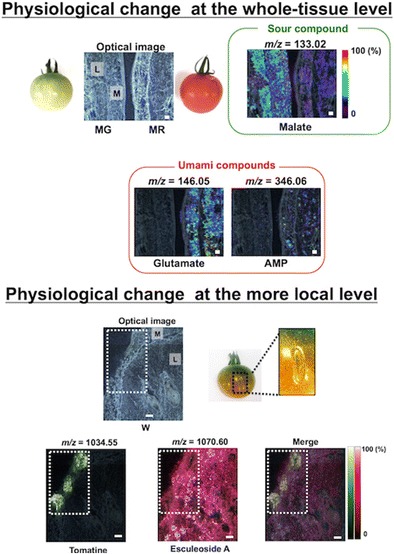
ᅟ

## Introduction

Endogenous metabolites are important components related to fruit phenotypes, such as color, flavor, taste, and texture. Their concentrations will dramatically and spatiotemporally change during growing and ripening processes [1–3]. Both biotic and abiotic stresses also trigger concentration changes, which lead to whole-tissue physiological changes, such as alterations in color and taste during the ripening process, or more local and partial responses, such as morphological changes induced by wounding and pest-associated stresses [4, 5]. These events are strictly regulated, and alterations in metabolic dynamics occur as the result of a wide range of biochemical processes, including enzymatic or non-enzymatic reactions [6–8].

The metabolome closely represents the phenotype of an organism under a given set of conditions and is defined as the “compound-level-phenotype” of the genomic information. Metabolomics is an effective approach for understanding global metabolic changes in fruit [9]. This approach allows for the quantitative determination of each metabolite and for tracing metabolic regulation in tissues during ripening and wounding stress. It can reveal potential relationships between metabolic signatures and phenotypes, such as physiological appearance and functional characteristics. These findings enhance the understanding of physiological mechanisms in the fruit, and this approach is expected to contribute to plant breeding and postharvest technology, such as food preservation and processing. Conventional metabolomics uses gas chromatography–mass spectrometry (GC–MS) and liquid chromatography–MS (LC–MS) to detect various metabolites, which appear at average levels, simultaneously in whole tissues. However, these methods do not address the spatial aspects of more local physiological phenomena, such as metabolic responses against wounding or pest stress at tissue micro-regional levels. Information on spatiotemporal metabolic behavior is quite important to precisely understand physiological changes and responses related to the tissue micro-regions of the functional compartments in fruit.

Matrix-assisted laser desorption/ionization–mass spectrometry imaging (MALDI–MSI) is a new remarkable technology [10–12]. This technique determines the spatial distribution of biomolecules, such as low-molecular-weight compounds, metabolites, proteins, and peptides, in tissue at a high spatial resolution without any labeling. Metabolite distributions were reported in several plant tissues, such as blueberry, cereal grains, and Arabidopsis [13–15]. However, sample preparation methods were different among plant tissues, and no observations focused on more local physiological responses in the tissue. Tomato (*Solanum lycopersicum* L.) fruit is an edible horticultural crop that is highly consumed worldwide and is used as a model plant of maturation [1]. Studies of tomato fruit, focused on understanding highly resolved metabolic behavior, are expected to unravel basic mechanisms of physiological changes and responses, as well as morphological alterations. However, the MALDI–MSI technique has not yet been applied in tomato fruit research. Here, we succeeded in determining the spatial distributions of multiple primary and secondary metabolites simultaneously within tissue sections of mature red (MR) tomato fruit using MALDI–MSI technique. Their unique localizations were observed in distinctive tissue compartments, such as mesocarp and locule regions. To investigate whole physiological changes at the metabolite level in the tomato fruit, metabolite distributions were compared using two different samples, mature green (MG) and MR tomato fruits. Furthermore, we assessed more local metabolic changes in tomato fruit during the ripening process after wounding, using MG as a control for the beginning stage of ripening and the pink stage (P) fruit, corresponding to the mid-stage. The wounded (W) fruit was prepared by wounding MG and at ripening stage up to the P. We compared the difference between MG and P to determine the metabolic changes occurred from the beginning to the mid-stage of ripening. Additionally, we investigated wound-specific metabolic alterations using the two types of P fruits between non-wounded (NW) and W. The MALDI–MSI technique enabled us to investigate large-scale and more local physiological responses of tomato fruit by detecting spatiotemporally resolved metabolic behaviors.

## Materials and methods

### Materials

Indium thin oxide (ITO)-coated slide glass was obtained from Matsunami Glass (Osaka, Japan). A matrix, 9-aminoacridine (9-AA), was purchased from Merck (Kenilworn, NJ, USA). Another matrix, 2,5-dihydroxybenzoic acid (DHB), and the organic solvents, internal standards, and metabolite standards were also obtained from Wako Pure Chemical Industries (Osaka, Japan).

### Preparation of tomato (*S. lycopersicum* L.) fruit

Tomato (*S. lycopersicum* L. ver. Chika) fruits were produced in Sotetsu horticultural farm (Kumamoto, Japan). We purchased both the mature green (MG) and mature red (MR) tomato fruits. MG and MR fruits were harvested at around 35 and 45 days after pollination, respectively. The wounded (W) sample was prepared by wounding MG fruits with the stainless blade. The wounding width was 5.0 mm and the depth was 1.0 mm. Both W and non-wounded (NW) samples were incubated on the room temperature for 1 week under non-sterile condition. Both W and NW samples were the same pink-stage fruits, corresponding to the mid-stage, in which less than 60% of the pericarp had changed from green to red during the ripening. All tomato fruits were stored at −20 °C.

### MALDI–MSI analysis of the tomato fruit section

The tomato fruit tissues were sectioned at 10 μm thickness using a cryomicrotome, and then the sections were thaw-mounted onto ITO-coated glass slide. In this process, we used the optical cutting temperature (O. C. T.) compound for directly mounting the whole tomato fruit on the stage of the cryomicrotome. For 9-AA-based MALDI–MSI experiment, matrix (9-AA) was sublimated using iMLayer (Shimadzu, Kyoto, Japan), a sample preparation device which can monitor temperature and matrix deposition thickness during the sublimation. The powder of 9-AA (600 mg) was sublimated at 220 °C under vacuum conditions (5 × 10^−2^ Pa) and the thickness of matrix was monitored at 0.5 μm. After sublimation, 10% methanol was vapored to promote both extraction of metabolites from tissue and recrystallization at 60 °C for 3 min [16]. For DHB-based MALDI–MSI experiments, matrix solution (20 mg/mL DHB in methanol containing 0.1% trifluoroacetic acid (TFA, *v*/*v*)) was sprayed with an airbrush in draft hood under room temperature. After matrix deposition, samples were subjected to MALDI–MSI measurements. MALDI–MSI was performed using single reflectron-type MALDI–time-of-flight (TOF)-MS (AXIMA Confidence, Shimadzu). Quadrupole ion-trap (QIT)-type (AXIMA QIT, Shimadzu) and TOF/TOF type (AXIMA Performance, Shimadzu) instruments were used for identification of metabolites by MS/MS analysis. These instruments were equipped with a 337-nm nitrogen laser, and all measurements were performed in both positive and negative ionization reflectron modes with 50 μm spatial resolution (ten laser shots/data point). The signals were collected between *m*/*z* = 80–890 (negative) or between *m*/*z* = 1000–1400 (positive). A number of peaks derived from metabolites were calculated by in-house script using Python (http://www.python.org). We first detected the peaks derived from the tomato fruit sample as sample peak, and non-tissue peaks on the glass plate were defined as blank peak. These peaks were exported as centroid format, and then isotope peaks were deleted. Of these peaks, sample peaks with the intensity over five times stronger than that of blank peak within the same *m*/*z* regions were considered as the net peaks derived from endogenous metabolites. Metabolites were identified or estimated by comparing MS/MS spectra with standard compounds or databases (MassBank, http://www.massbank.jp; METLIN, http://metlin.scripps.edu). Acquired MSI data were processed with a freely available software Biomap (http://ms-imaging.org/wp/). This software was used for creation of two-dimensional ion-density maps, normalization of peak intensity, adjustment of the color scale, and quantification of the ion intensity. All data was merged between optical and ion images.

### Quantitative analysis of metabolites with LC–triple quadrupole (QqQ)–MS

The pericarp (epicarp and mesocarp) and locule fractions (100–200 mg) were homogenized in 80% methanol, including 5 μM 10-camphorsulfonic acid (10-CS) for evaluating the extraction efficiency, on ice using dounce tissue grinders. After centrifugation at 15,000×*g* for 10 min, the supernatant was collected. These samples were filtered using 0.22 μm PTFE filter (EMD Millipore, Billerica, CA, USA). The filtered samples were analyzed by high-performance liquid chromatography with triple quadrupole (LC–QqQ)–MS (LC8040, Shimadzu, Kyoto, Japan). The instrument was fitted with a Synagi Hydro-RP column (C18, 100 mm × 2.0 mm i.d., Phenomenex, Torrance, CA, USA), ovened at 40 °C. Mobile-phase conditions were as follows: linier gradient analysis with mobile-phase A, 10 mM tributylamine (TBA)/15 mM acetic acid in ultra pure water, and mobile-phase B, methanol. After a 3-min isocratic run at 100% of eluting solvent A, the ratio of eluting solvent B was linearly increased to 40% from 3 to 5 min and to 90% from 5 to 7 min. The composition of 90% of the eluting solvent B was maintained for 5 min. For the MS, the instrument was operating an electrospray ionization source in both positive and negative ionization modes. The ionization parameters were performed under the following conditions: capillary voltage, 4.5 and −3.5 kV; the nebulizer gas flow, 1.5 L/min; the CDL temperature, 250 °C; and heatblock temperature, 400 °C. Multiple reaction monitoring mode with a dwell time of 2 s per channel were used for the targeted analysis of ten metabolites. Identified metabolites were quantified using authentic standards, and then the data were expressed as the ratios (locule/epicarp) shown in Fig. S3 in the Electronic Supplementary Material (ESM).

### Heat map analysis

We used heat map analysis to determine the certainty of the present 9-AA-based MALDI–MSI results by comparing MSI relative peak intensities in both locule and mesocarp regions with LC–MS-quantified data on both locule and pericarp (epicarp and mesocarp) regions in MR tomato fruit. In the MSI experiment, we focused on both locule and mesocarp regions. Regions of interest (ROIs), indicated in Fig. S3B (see ESM), were established in a part of homogenous-tissue regions on the optical image of MR tomato fruit by using BioMap software. Each average intensity from averaged mass spectrum of either locule or mesocarp region determined within selected ROIs. The data was converted into the ratio of locule to mesocarp region. In LC–MS experiments, the epicarp could not be perfectly separated from the mesocarp using fresh fruit. Therefore, we used both mesocarp and pericarp fractions as a crude mesocarp fraction, even though it includes a small amount of epicarp. The metabolite concentration data of LC–MS was converted into the ratio of locule to pericarp region. We compared the ratios between MALDI–MSI and LC–MS using the heat map analysis. The ratio of ten common metabolites was visualized with MultiExperiment Viewer (http://www.Tm4.org). In this experiment, we used three replicate data for LC–MS analysis and four replicate data for MALDI–MSI analysis. These averaged values were subjected to the heat map analysis.

### Evans Blue staining

Fresh wounding tomato fruit including wound region cut using a razor blade were stained with 0.5% (*v*/*v*) Evans Blue solution (Wako Pure Chemical Industries) partially modified from previous report [17]. After 5 min, the fruit was washed with distilled water and put on the slide glass. This sample was subjected to light microscope (AS-3100, Microadvance, Osaka, Japan). Non-viable cells had blue coloration and viable ones were not stained.

## Results

### MALDI–MSI-based visualization of spatial distributions of metabolites in MR tomato fruit

**Fig. 1:**
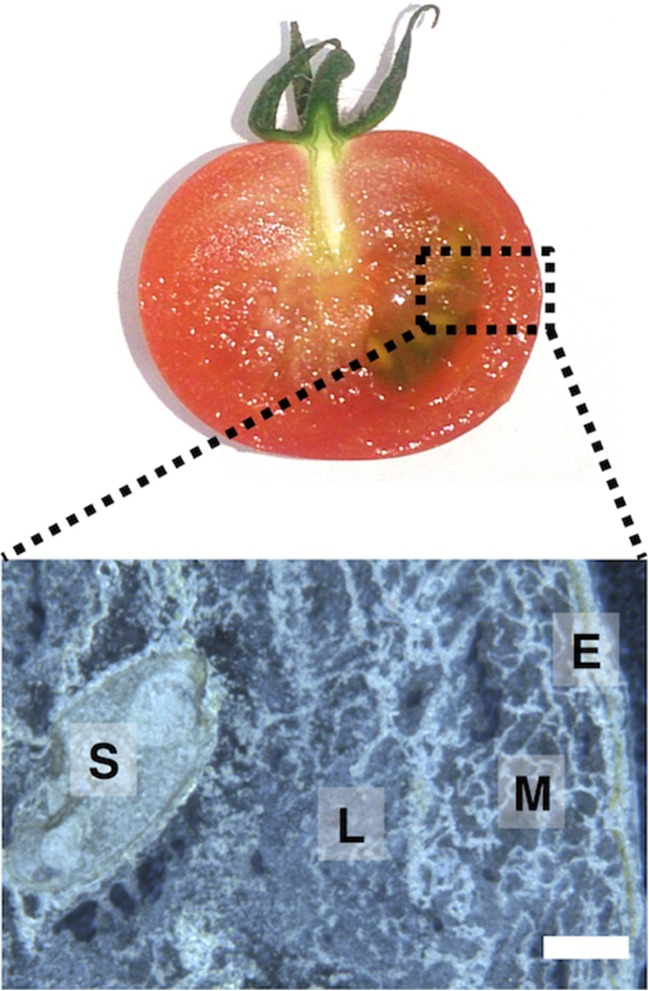
Thin-section of mature red (*MR*) tomato fruit using cryomictorome without any embedding materials. *E* epicarp, *M* mesocarp, *L* locule, *S* seed. *Scale bar* = 1.0 mm

**Fig. 2:**
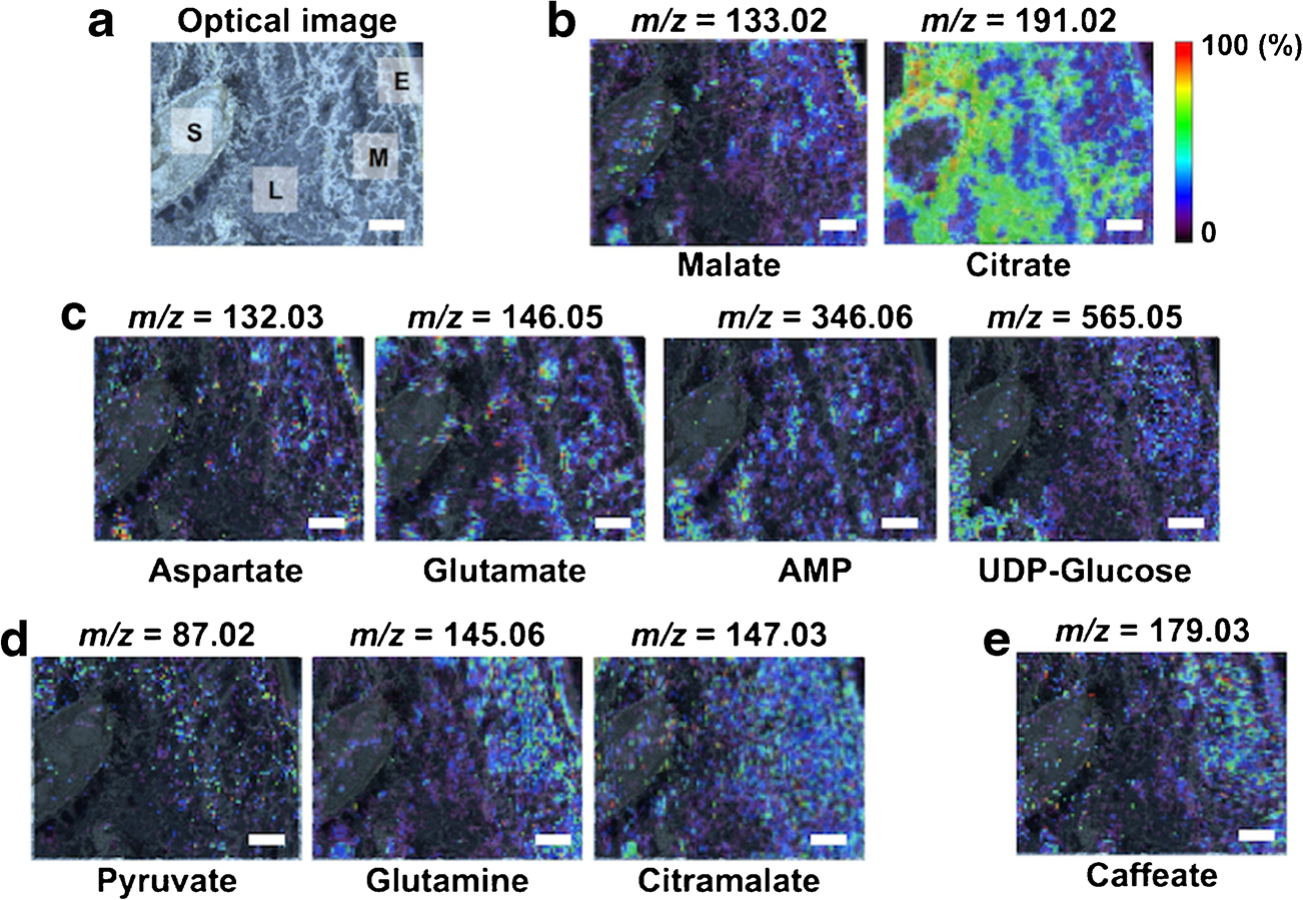
In situ metabolite MS imaging of the MR tomato fruit. **a** Optical image of thin-section of the MR tomato fruit. *E* epicarp, *M* mesocarp, *L* locule, *S* seed. Ion images of **b** sour compounds, **c** umami compounds, **d** primary metabolites, and **e** secondary metabolite, respectively. *Scale bar* = 1.0 mm

To perform MALDI–MSI experiments, solid samples are thinly sectioned and mounted onto a conductive stage. In this study, we prepared the thin-sections of frozen fruit using a cryomicrotome without any embedding materials, and then the sections were mounted onto indium tin oxide (ITO)-coated glass slides using the thaw-mounting method. The shape of each organ structure, including epicarp, mesocarp, locule, and seed, was maintained in the cryosections (Fig. 1). The choice of the matrix for MALDI–MSI is an important point for metabolite imaging. Here, we used 9-aminoacridine (9-AA) because it is a superior MALDI matrix for ionizing a broad range of negatively charged metabolites, such as nucleotides, cofactors, phosphorylated sugars, amino acids, lipids, and carboxylic acid [18]. The choice of matrix deposition method is also important for achieving successful MSI results with high sensitivity and high spatial resolution. Here, we used a method of sublimation coupled with recrystallization because it was highly reproducible and highly sensitive, and it eliminated the migration of analyte compounds during matrix deposition [16, 19, 20]. The tissue sections of MR tomato fruit were subjected to the matrix deposition and subsequent MALDI–MSI measurement. In total, 91 peaks derived from endogenous metabolites were detected (ESM Fig. S1), and 34 of these peaks had unique distributions in the MR tomato fruit (ESM Fig. S2). Both the localization and intensity of the peaks were different among the four tissue compartments (epicarp, mesocarp, locule, and seed). Among these, we successfully identified ten metabolites, including primary metabolites (sour compounds and umami compounds) and secondary metabolite (caffeate), by comparisons with MS/MS spectra of standard compounds (Fig. 2, ESM Table S1).

MALDI–MSI provides useful information on the distribution of biomolecules over the two-dimensional analytical regions. However, the signal intensity information does not reflect the “absolute” amounts but the “relative” amounts of biomolecules. In some cases, the resultant images do not reflect the relative amount of an analyte in different regions of the same sample because of the differences in the matrix effects, the ion suppressing or enhancing effect, between different regions [21, 22]. We determined the certainty of the present MSI results by comparing MSI relative peak intensities in both locule and mesocarp regions with LC–MS-quantified data on both locule and pericarp (epicarp and mesocarp) regions in the MR tomato fruit. Although, MSI detected the metabolite distributions in the mesocarp and locule regions, but they were hardly confirmed in the epicarp and seed regions. In this experiment, we focused on the locule and mesocarp regions.

Each average intensity of both representative locule and mesocarp regions determined within a selected regions of interest (ROIs) (ESM Fig. S3). The data was converted into the ratio of locule to mesocarp region. In LC–MS experiments, the epicarp could not be perfectly separated from the mesocarp using fresh fruit; therefore, we used both locule and pericarp fractions as crude mesocarp fractions, even though it included a small amount of epicarp. The metabolite concentration data was converted into the ratio of locule to pericarp region. We compared the ratios between MALDI–MSI and LC–MS data using the heat map analysis. This analysis showed relatively similar tendencies for most metabolite levels, although the values were different in the MSI and LC–MS data (ESM Fig. S3). These results showed, for the first time, that the present MALDI–MSI technique was able to visualize micro-region-specific distributions, with relatively quantitative values, of multiple metabolites simultaneously in the MR tomato fruit.

### Analysis of metabolic alterations using two different ripening tomato phenotypes

**Fig. 3:**
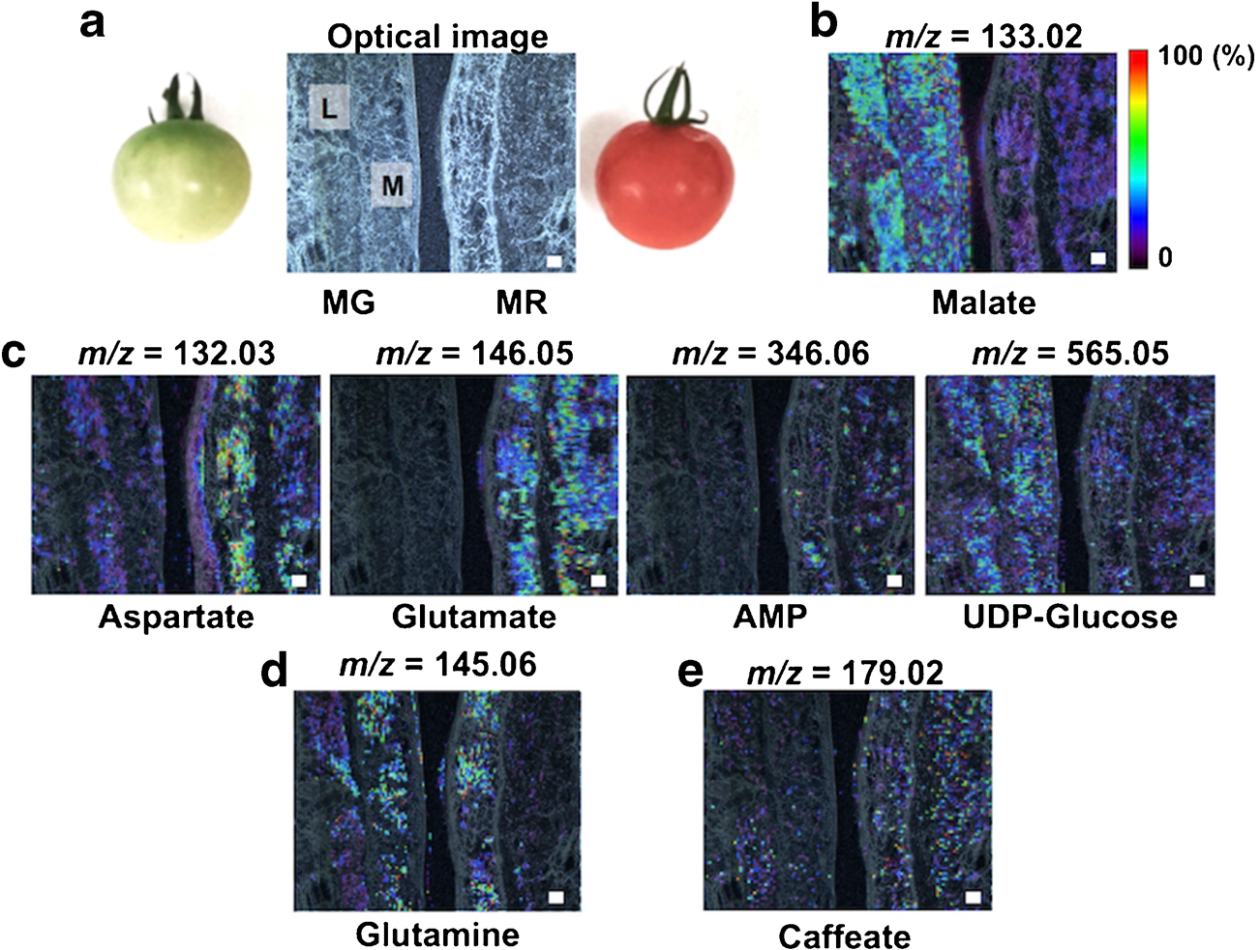
Analysis of the metabolic changes during the progression of ripening process using different ripening phenotypes with 9-AA-based MALDI–MSI. **a** Optical image of thin-sections of the mature green (*MG*) and MR tomato fruits. *M* mesocarp, *L* locule. Ion images of **b** sour compound, **c** umami compound, **d** primary metabolites, and **e** secondary metabolite. *Scale bar* = 1.0 mm

The concentrations of some taste-related metabolites, such as amino acids and organic acids, change dramatically at the whole-tissue level in tomato fruit during the ripening process [23]. Additionally, ethylene, a phytohormone, plays a key role in triggering fruit maturation [1–3]. However, the potential relationships between the presence of metabolites at the tissue micro-regional level and ethylene-induced fruit ripening still remains unclear. Here, we successfully visualized the spatial distribution of multiple metabolites in the MR tomato fruit. To further understand physiological property-related spatiotemporal metabolic alterations, we performed MALDI–MSI experiments using two tomato fruits, MG and MR, of different maturity (Fig. 3). The thin-sections of MG and MR were thaw-mounted onto the same conductive glass slide to compare the distributions of multiple metabolites in a single run, and these samples were subject to MALDI–MSI measurement. Metabolite distributions in the mesocarp and locule regions were observed, but they were barely seen in the epicarp and seeds. The amounts of umami compounds, glutamate (*m*/*z* = 146.06 [M–H]^−^) and adenosine monophosphate (AMP) (*m*/*z* = 346.06 [M–H]^−^), were increased in both regions of the MR fruit. Aspartate (*m*/*z* = 132.03 [M–H]^−^), another umami compound, was increased only in a mesocarp region. In contrast, malate (*m*/*z* = 133.02 [M–H]^−^), a constituent of sourness in immature fruit, had decreased compared with in MG fruit. However, the intensities of a secondary metabolite, caffeate (*m*/*z* = 179.02 [M–H]^−^), and other primary metabolite, glutamine (*m*/*z* = 145.05 [M–H]^−^), were similar in the analytical regions of both MG and MR samples (Fig. 3). Although there were differences in spatial resolutions, these local metabolic behaviors were in good accordance with the results of whole-tissue extractions from both ripening phenotypes, MG and MR, as previously reported by GC–MS and LC–MS [24]. Using MALDI–MSI, we succeeded in analyzing the spatiotemporal metabolic alterations in both mesocarp and locule regions during the ripening process of tomato fruit.

### Spatiotemporal metabolic visualization of local physiological responses to ripening and wounding stress

**Fig. 4:**
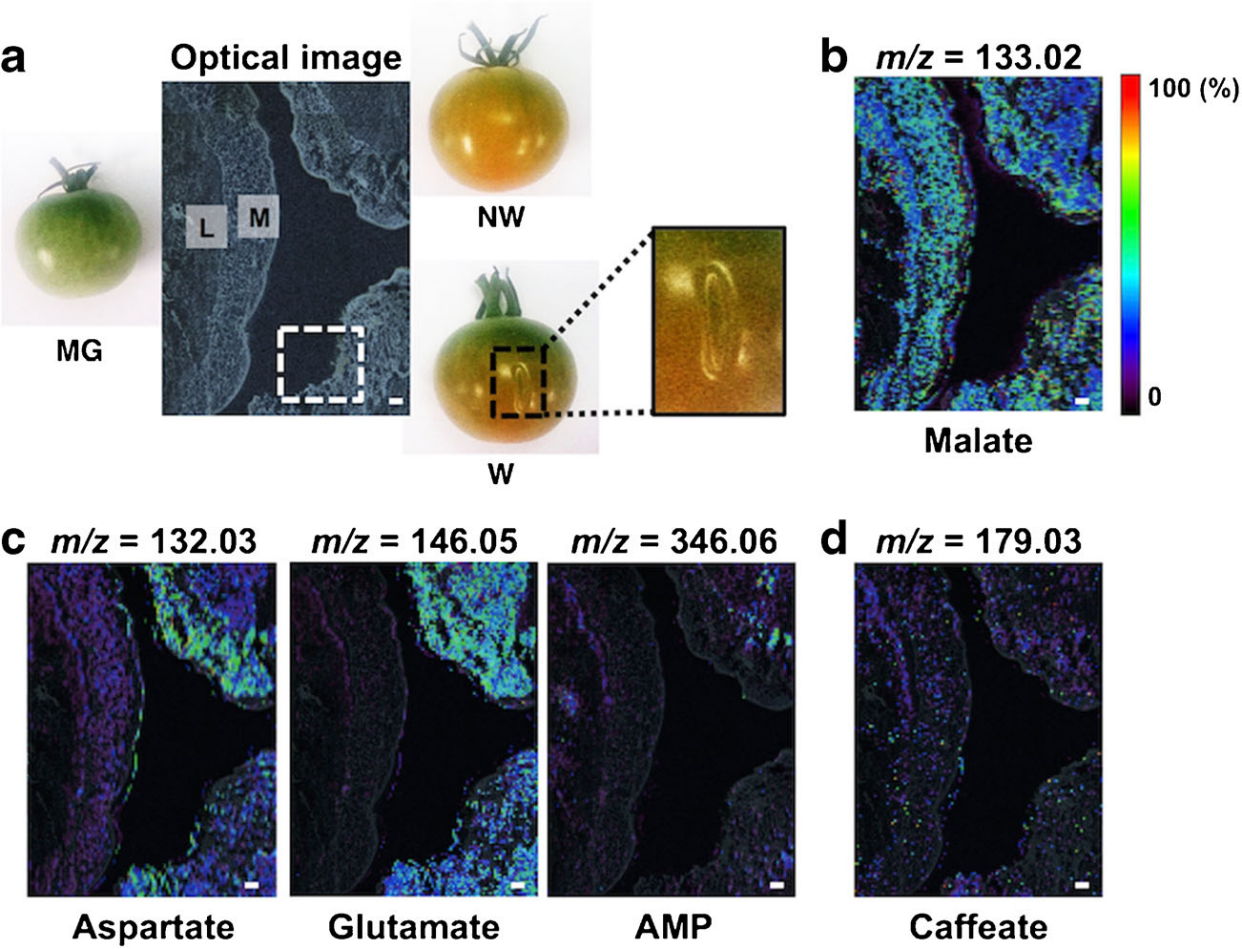
Analysis of metabolic alterations in the wounding stress in the tomato fruit with 9-AA-based MALDI–MSI. **a** Optical image of thin-sections of MG, non-wounded (*NW*), and wounded (*W*) tomato fruits. Both NW and W fruits in pink stage, corresponding to the mid-stage of ripening. *M* mesocarp, *L* locule. The *white square* indicates the wound region in tomato tissue Ion images of **b** sour compound, **c** umami compounds, and **d** secondary metabolite. *Scale bar* = 1.0 mm

**Fig. 5:**
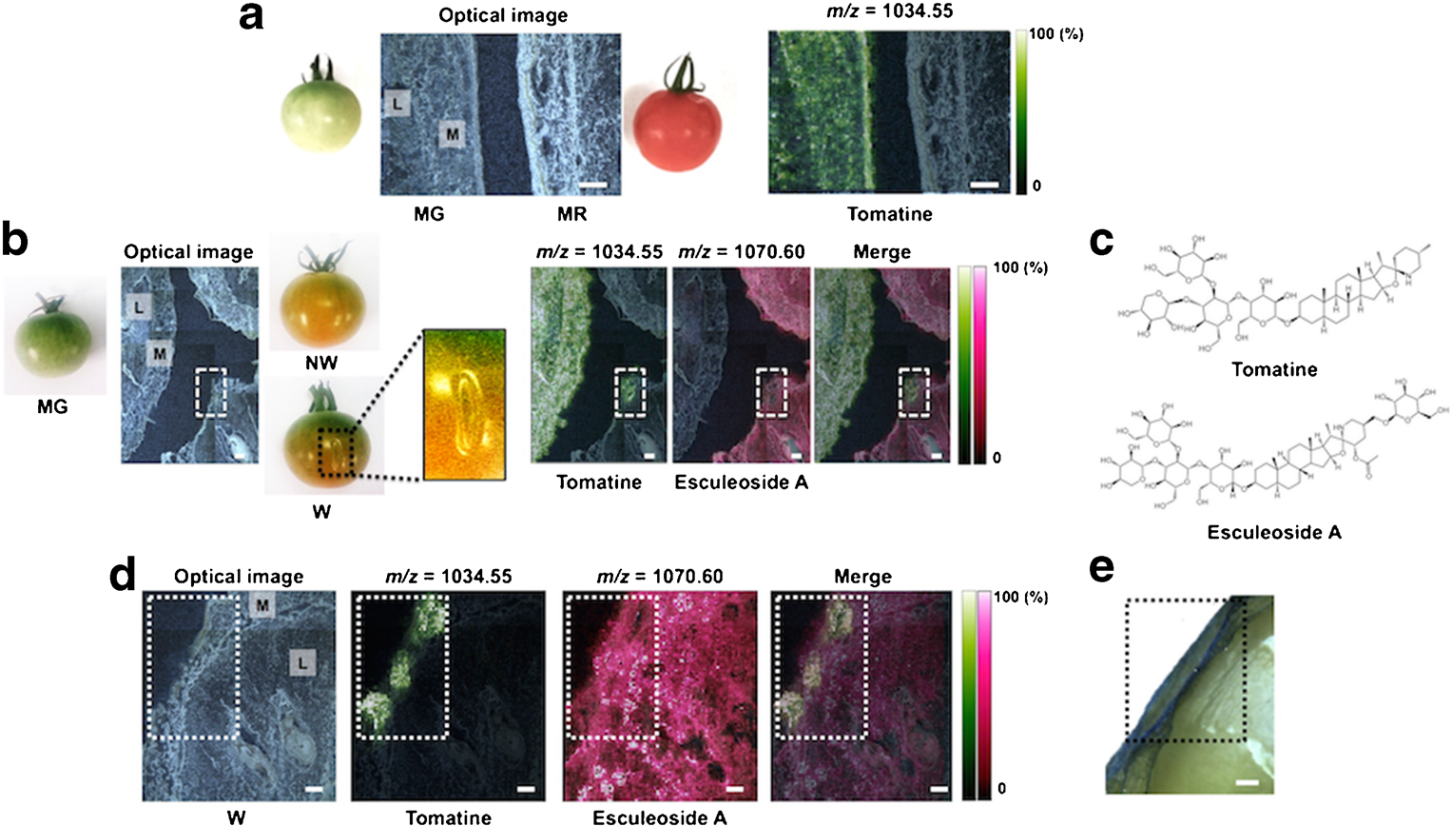
Visualization in more local physiological responses to ripening and wounding stress with DHB-based MALDI–MSI. **a** Optical image and ion image of tomatine in thin-sections of between MG and MR tomato fruits. *M* mesocarp, *L* locule. **b** Optical image and ion images derived from tomatine-related compounds among three phenotypes of tomato fruits, MG, non-wounded (*NW*), and wounded (*W*). Both NW and W fruits in pink stage, corresponding to the mid-stage of ripening. **c** Structures of tomatine (*m*/*z* = 1034.55 [M + H]^+^) and esculeoside A (*m*/*z* = 1270.60 [M + H]^+^), a glycosylated tomatine product. **d** Optical image and magnified ion images derived from tomatine-related compounds focused on the wound region. **e** Evans Blue staining for viability of the cells around wound region in tomato fruit. *Blue*-*colored area*; non-viable cells. The *broken box*; wound region. *Scale bar* = 1.0 mm

Plants produce ethylene during wounding stress, and it triggers various genetic and metabolic responses, but the details of these response mechanisms are not fully understood [4, 5]. In particular, local responses near the wound region are still unknown because conventional methods have not been able to assess the cooperative behaviors of multiple biomolecules at the micro-regional level. Here, a 9-AA-based MALDI–MSI analysis was performed to examine metabolic changes around the wound region in the tomato fruit during the ripening process after wounding. We used MG as a control for the beginning stage of ripening and the pink-stage (P) fruit corresponding to the mid-stage, in which less than 60% of the pericarp had changed from green to red during the ripening. The wounded sample (W) was prepared by wounding the MG tomato and at ripening stages up to the P stage. The non-wounded fruit at the corresponding ripening stages were defined as NW. We focused on the locule and mesocarp regions among MG, NW, and W fruits. First, we compared the difference between MG and P to determine the metabolic changes that occurred from the beginning to the mid-stage of maturation. In addition, we determined the differences in the metabolite distributions between NW and W fruits to investigate wound-specific metabolic alterations. As shown Fig. 4, an increase in the amount of glutamate and AMP, umami compounds, in P (NW and W) was observed in the local region as compared with MG. However, clear differences between NW and W were not observed among wound-specific metabolic responses to wounding. Next, we used the spray-coating method with 2,5-dihydroxybenzoic acid (DHB) as the matrix to detect other metabolites in the positive ionization mode. DHB-based MALDI–MSI detected tomatine (*m*/*z* = 1034.55 [M + H]^+^), a glycoalkaloid, in MG tissue, and it especially accumulated in both locule and mesocarp regions (Fig. 5a, c). In contrast, the same tomatine distribution was not observed in MR tissue. In addition, there was no accumulation of tomatine at the mid-stage of the ripening process, P (NW) (Fig. 5b). Thus, tomatine was lower during the ripening process. In contrast, a putative tomatine-glycosylated metabolite, esculeoside A (*m*/*z* = 1270.60 [M + H]^+^) [25], was observed in P, but there was no accumulation in MG (Fig. 5b, c). This tendency was inversely correlated with the presence of tomatine. Additionally, a magnified MSI data showed that, regardless of the mid-stage, tomatine accumulated in the wounded regions of W tissues (Fig. 5d). Esculeoside A showed an inverse presence in the same region. Evans Blue staining revealed cell death around the wound region, which was the representative physiological response against wounding stress (Fig. 5e). Thus, an alteration in the metabolism of tomatine may be caused by wound-induced cell death. These findings indicate that the MALDI–MSI technique is an effective approach for determining the spatially resolved metabolism of a glycoalkaloid, tomatine, based on local responses to the ripening process and wounding stress.

## Discussion

In plants, the production of endogenous metabolites is strictly regulated by the complicated signal transduction pathways of the various organs at each growth stage. In particular, there are dynamic changes in the levels of primary and secondary metabolites during developmental processes, such as seed germination, flowering, ripening, and organ abscission [26, 27]. The climacteric stage of fruit ripening process in tomato and banana (*Musa* spp.) is associated with the increased production of ethylene and a brief increase in cellular respiration. Ethylene is necessary to trigger fruit ripening and senescence processes, but the interaction of ethylene with other signaling pathways is not fully understood [28]. Thus, elucidating the potential relationships among ethylene-related events and metabolic dynamics during the maturation processes in fruit improves our understanding of the mechanisms responsible for phenotype formation, including color, flavor, taste, and morphology. Recently, the MALDI–MSI technique has received increasing attention as an attractive method to determine the spatial distribution of biomolecules in plant materials, such as blueberry, rice grain, and Arabidopsis leaves [13–15]. Here, we applied a 9-AA-based MALDI–MSI technique to elucidate the spatial distribution of metabolites in complex tomato fruit structures (epicarp, mesocarp, locule, and seed). We were able to visualize, for the first time, the distribution of multiple primary and secondary metabolites simultaneously within tissue sections of MR tomato fruit in a single run. The concentration of an organic acid, malate, at the whole-tissue level gradually increases at the early growth stage, and then it decreases as the ripening process progresses in tomato, while another organic acid, citrate, increases during the late developmental stages [29]. However, little is known about their alterations at tissue micro-regional levels. To reveal metabolite spatiotemporal alterations, including those of the associated organic acids, we performed MALDI–MSI experiments using MG and MR fruits, which were at different stages of maturity. The amount of glutamate and AMP, umami-related compounds, increased in both the mesocarp and locule compartments of the fruit tissue during the ripening progression from MG to MR, while malate, a sour compound, decreased in both regions (Fig. 3). Generally, the pericarp’s color and taste, such as sour, sweet, and umami, dramatically change during the ripening process. They are related to metabolic alterations, such as the accumulation of carotenoids and amino acids, and ethylene effects, such as the decrease of organic acids [1, 2]. When combined with the whole-fruit level findings, our data provide evidence of new relationships between such phenotypes and spatially resolved metabolic changes during the ethylene-induced progression of ripening in the tomato fruit.

The MALDI–MSI analyzed local metabolic responses against wounding stress and determined the spatial distributions of both tomatine and its glycolated metabolite, esculeoside A, in the tomato fruit (Fig. 5). Tomatine is a glycoalkaloid and is involved in host defenses against phytopathogens by inhibiting their activities [30]. In addition, plants protect themselves by releasing phytoalexin, an antibacterial agent, when they are attacked by pests. Immature green fruit has a high concentration of tomatine in the epicarp region, but its concentration decreases in the MG fruit [31, 32]. However, the level of tomatine was still unknown in the P stage, which corresponds to the mid-term of the ripening process. In this study, the localization of tomatine was determined in fruit at three different developmental stages (MG, P, and MR). In addition, its tomatine-glycolated product, esculeoside A, showed an inverse presence with tomatine between the MG and P. Alterations of tomatine and esculeoside A were present in regions where cell death was induced by wounding. This is the first report demonstrating a spatially resolved metabolic alteration of a glycoalkaloid based on physiological changes and responses, such as ripening and wounding, respectively. The existence of genes and proteins concerned with biosynthesis of tomatine in tomato fruit have been reported [33, 34], but there were few observations of gene and protein expression levels associated with the glycolation pathway of glycoalkaloid, even though the tomato genome has been sequenced [35]. Thus, the glycolation mechanism from tomatine to esculeoside A was still unclear. Our findings implicate cell death-related biochemical events in the alterations of tomatine and esculeoside A. A better understanding of this new relationship will shed light on the regulatory mechanisms of glycoalkaloids and their biological significance in local physiological responses against wound-related stress.

In MALDI–MSI, spray-coating method with an airbrush has been used to apply the matrix solution on sample tissues because it is a simple and convenient technique. Thus, DHB-based MALDI–MSI was performed to visualize the distribution of tomatine-derived compounds with high sensitivity using this method (Fig. 5a–c). In particular, this technique determined the spatial distribution of esculeoside A with a higher sensitivity than the sublimation coupled with recrystallization method (data not shown). The spray-coating technique often requires manual operation, and the quality of the resultant sample is completely dependent on the user’s skill. In some cases, this method has shown poor reproducibility [36]. Here, sublimation and its combination with recrystallization method was used as the matrix deposition strategy. Sublimation was previously reported to be suitable for the detection of glucosinolates in Arabidopsis leaves [15]. The application of the matrix, and the monitoring of its thickness, is performed by fully automated equipment, which is easily operated, leading to a low human error. Furthermore, the sublimation method is suitable for the high spatial resolution imaging when compared with the spray-coating method [37]. However, the coverage and sensitivity of detectable metabolites were different in the two methods [36]. In addition to our attempts, considering these circumstances, researchers must select the matrix deposition method suitable for detecting the metabolites of interest.

Using the method of sublimation coupled with recrystallization to apply 9-AA as a matrix, we detected more than 90 peaks derived from endogenous metabolites in the tomato fruit (ESM Fig. S1). In addition, we analyzed 9-AA-deposits in tissue samples by sublimation alone, but the numbers and intensities of the peaks derived from endogenous metabolites were lower than those of sublimation coupled with recrystallization (data not shown). In this study, most of the detected peaks could not be annotated (Fig. 2, ESM Table S1). The metabolites derived from sample tissue sections were generally identified and/or estimated by comparing MS/MS spectra with those of standard compounds or public databases. However, it is difficult to theoretically annotate compounds for which standard MS/MS spectra or database information is not available. To overcome this problem, we are now developing a methodology of standard compound-independent determinations of elemental compositions using ultrahigh-resolution MS [38, 39]. In addition, optimizing multiple faces of the experimental conditions, including matrix selection, matrix synthesis, and matrix application methods, are required to improve the sensitivity and coverage of detectable metabolites using the MALDI–MSI technique. Improving these factors may help to elucidate the complex mechanisms of physiological changes and responses by visualizing detailed metabolic alterations at tissue micro-regional levels and link them with given phenotypical characteristics.

## Conclusions

Conventional metabolomics approaches are effective approaches for understanding global metabolic alterations in plant tissue. However, these techniques do not address the spatial aspects of more local physiological phenomena, such as metabolic responses against wounding or pest-associated stresses at tissue micro-regional levels. To elucidate the distribution of metabolites in the complex structures of tomato fruit, we were able to visualize, for the first time, the spatial distribution of multiple primary and secondary metabolites simultaneously within tissue sections of MR tomato fruit using 9-AA-based MALDI–MSI combined with the matrix sublimation/recrystallization method. Moreover, we performed a MALDI–MSI experiment using MG and MR fruit, which are at different maturity stages, to further understand the physiological properties related to spatiotemporal metabolic alterations. The amount of umami compounds was high in both locule and mesocarp regions during the ripening process. In contrast, the level of sour compounds was low in both regions. Furthermore, DHB-based MALDI–MSI was applied to evaluate micro-region-specific metabolic alterations in response to wounding stress. We succeeded in visualizing alterations between tomatine and its glycosylated product, esculeoside A, in the wound region. These new findings suggest that our MSI technique will contribute to an enhanced understanding of physiological alterations of tomato fruits, and may lead to the creation of new plant breeding and postharvest technology.

## Electronic supplementary material

Below is the link to the electronic supplementary material.

ESM 1 (PDF 514 kb)
