# Supplementary material for: Spatially resolved metabolic distribution for unraveling the physiological change and responses in tomato fruit using matrix-assisted laser desorption/ionization–mass spectrometry imaging (MALDI–MSI)
Source: Anal Bioanal Chem. 2016 Dec 8;409(6):1697–706. doi: 10.1007/s00216-016-0118-4 (PMC5306346; doi:10.1007/s00216-016-0118-4)
Supplement: Supplementary file 1 — (PDF 514 kb) [file 216_2016_118_MOESM1_ESM.pdf]

Analytical and Bioanalytical Chemistry

Electronic Supplementary Material

Spatially resolved metabolic distribution for unraveling the physiological change and responses in tomato fruit using matrix-assisted laser desorption/ionization–mass spectrometry imaging (MALDI–MSI)

Junya Nakamura, Tomomi Morikawa-Ichinose, Yoshinori Fujimura, Eisuke Hayakawa, Katsutoshi Takahashi, Takanori Ishii, Daisuke Miura, Hiroyuki Wariishi

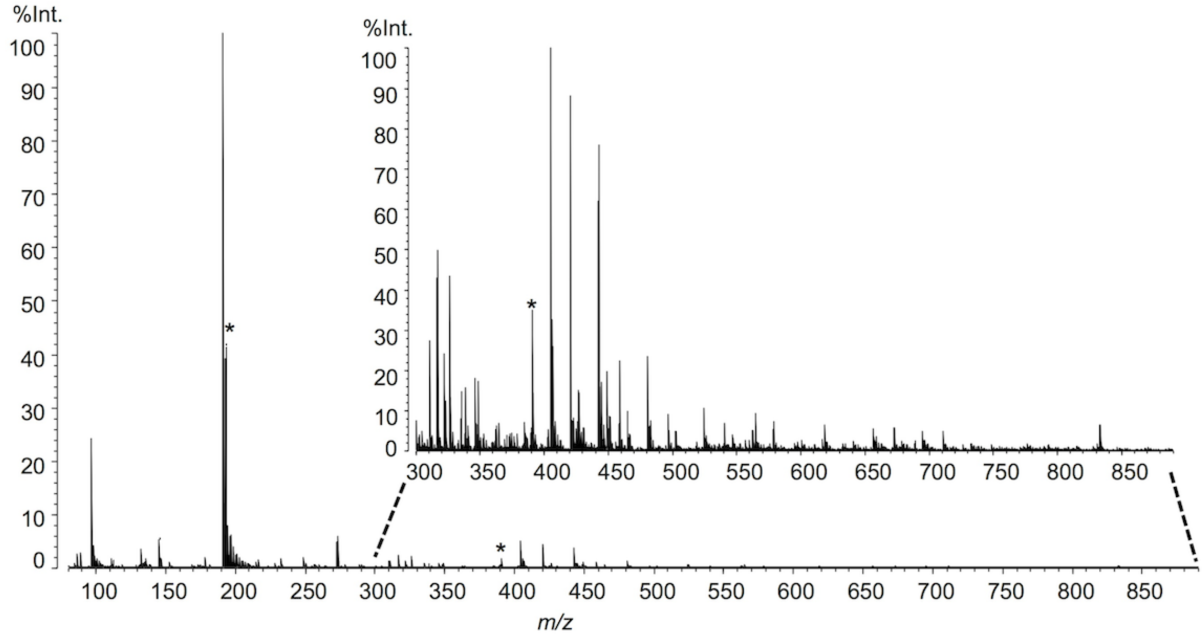

Fig. S1 Overview of the averaged mass spectrum acquired from the direct analysis of MR tomato fruit thin section. Whole averaged mass spectrum acquired from MR tomato thin-sections (*m/z* = 80-890). Enlarged spectra from whole averaged mass spectra data (*m/z* = 300-890). Asterisk marks; matrix-derived peaks (matrix: 9-AA)

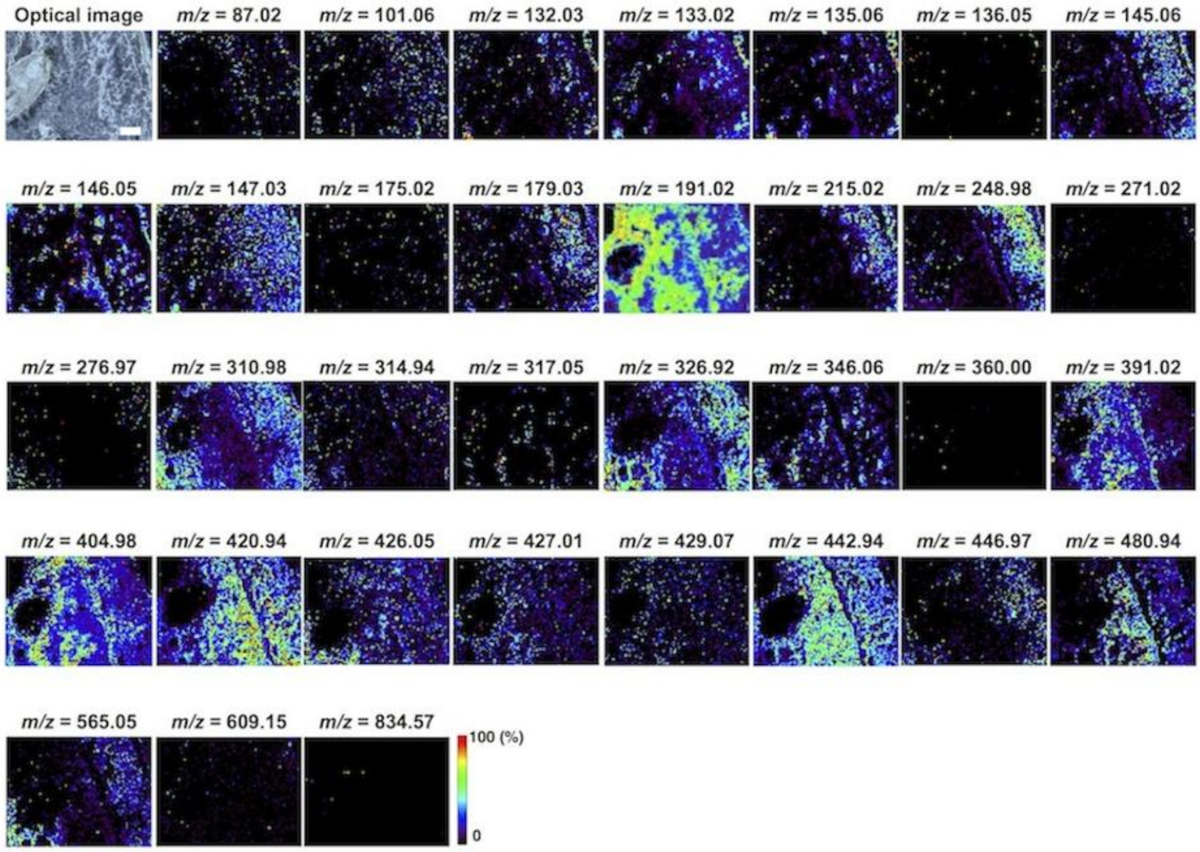

Fig. S2 In situ compounds imaging of MR tomato fruit section. Visualization of unique distributions of 34 compounds derived from metabolites in the MR tomato fruit section. Scale bar = 1.0 mm

Table S1 Mass-to-charge ratio, molecular species and fragmentations observed on the tomato fruit section

m/z	*Molecular species*	*Fragments observed*	*Formula*
87	pyruvate	44	$C_3H_4O_3$
132	aspartate	115, 88	$C_4H_7NO_4$
133	malate	115	$C_4H_6O_5$
145	glutamine	127	$C_5H_{10}N_2O_3$
146	glutamate	128, 102	$C_5H_8NO_4$
147	citramalate	129, 101, 87	$C_5H_8O_5$
179	caffeate	135	$C_9H_8O_4$
191	citrate	173, 111	$C_6H_8O_7$
346	AMP	211, 151, 97	$C_{10}H_{14}N_5O_7P$
565	UDP-glucose	403, 323	$C_{15}H_{24}N_2O_{17}P_2$

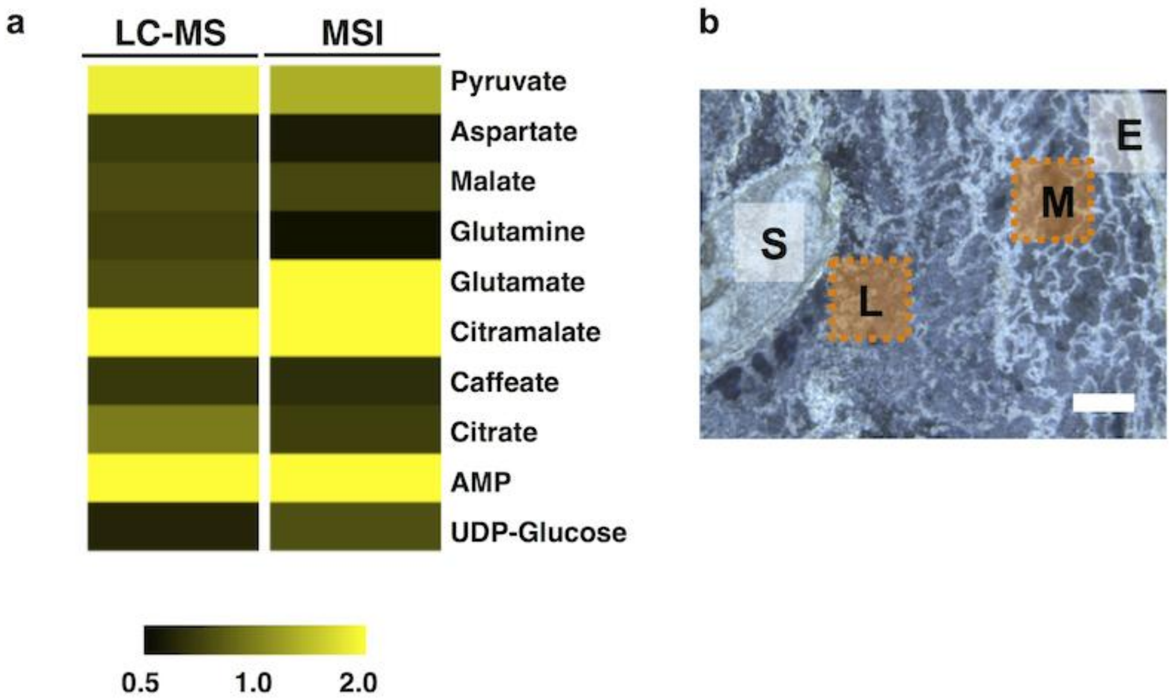

Fig. S3 A comparison of the average concentration/intensity between the whole tissue regions and partial tissue regions. **a** In the heat map, 10 common metabolites ratio detected by LC–MS and MSI. The ratio of the average intensity (locule/pericarp) in LC–MS data (left column). The ratio of the average intensity (locule/mesocarp) obtained from ROIs (L) and (M), which are indicated in the panel **b**, in MALDI–MSI data (right column). **b** Optical image of thin-section of the MR tomato fruit.

E; epicarp, M; mesocarp, L; locule, S; seed. Scale bar = 1.0 mm

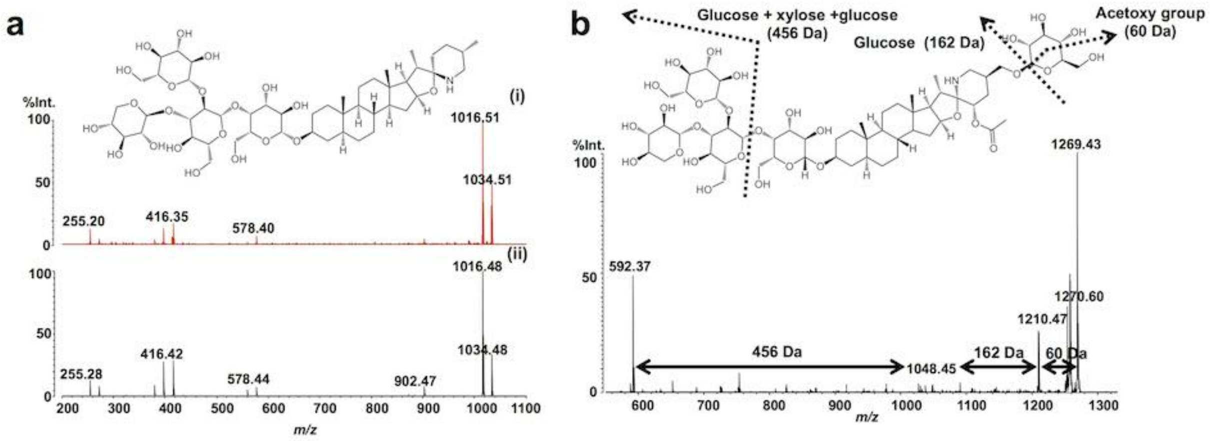

Fig. S4 Identification or estimation of compounds derived from glycoalkaloids by comparing with MS/MS data. **a** Identification of MS peaks of *m/z* = 1034.55 ($[M+H]^+$) as tomatine by comparing with MS/MS spectra between tomato fruit section and standard compound. (i) The MS/MS spectrum acquired from the tomato fruit section. (ii) The MS/MS spectrum of tomatine acquired from standard sample. **b** Estimation of MS peaks of *m/z* = 1270.60 ($[M+H]^+$) as esculeoside A by MS/MS data. The fragments were referred from [25]
